# The Epidermolysis bullosa Center Freiburg – patient care, diagnostics and research

**DOI:** 10.1186/1750-1172-9-S1-O1

**Published:** 2014-11-11

**Authors:** Leena Bruckner-Tuderman, Daniela Kirstein

**Affiliations:** 1 Department of Dermatology, Medical Center - University of Freiburg, 79104 Freiburg, Germany

## 

**Figure 1:**
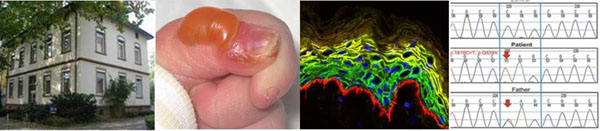
Panels from left to right – Building of the EB Center Freiburg; skin blister in EB; immunofluorescence staining of the skin; mutation analysis

The Epidermolysis bullosa (EB) Center Freiburg is the coordination center of the German EB-Network and a national center of excellence for rare skin fragility disorders. It combines clinical activities with internationally competitive research and deals with molecular diagnostics, clinical management, elucidation of disease mechanisms, and development of evidence-based novel therapies (Figure 1). EB is a group of severe and socially relevant genetic skin diseases characterized by mechanically induced blistering and life-long fragility of the skin. EB has a highly negative personal, medical and socio-economic impact on the life of the patients and their families, and the unmet medical need is high. - The activities of the EB-Network build upon the combination of clinical and scientific expertise of the partners in Germany and neighboring countries (cross-border care), and upon the synergies generated in the past years.

The EB Center Freiburg performs molecular diagnostics, coordinates multidisciplinary care for patients and their families, advices general practitioners, medical specialists, nursing staff and therapists, and disseminates information to lay public and media. The office of the Center is available for enquiries for 24 hours and responds within 24 hours. The team includes a coordinator, physicians, nurses, a social worker, a documentary clerk, scientists and laboratory technicians with expertise in EB. The consultations are usually out-patient or day clinic appointments, but hospital admission is possible for severe cases requiring extensive medical treatments. Standardized clinical practice with a diagnostic algorithm and standardized patient documentation facilitates diagnostic processes, and a weekly EB-expert meeting evaluates all diagnoses as a quality assurance measure. Currently the EB-patient registry contains data of >1000 patients with molecular genetic diagnosis and has an associated biomaterial collection of skin biopsies, cells and blood samples. These serve as basis for research on epidemiology of EB and for clinical and laboratory investigations on novel causes, disease mechanisms, genotype-phenotype correlations and treatments for EB. - In addition to numerous international research collaborations, the EB Center is actively involved in larger structures for rare diseases. The Freiburg Center for Rare Diseases [http://www.uniklinik-freiburg.de/fzse.html] provides high-level scientific expertise, innovative diagnostics and interdisciplinary care for people with rare disorders of the skin, the musculoskeletal system, the kidney, the lung, the eye, the blood and the immune systems. Internationally, EB-CliNet [http://www.eb-clinet.org], a European network of EB Centers, and the Genodermatoses Network, an international network on rare skin diseases for professionals and patients [http://www.genodermatoses-network.org] aim at establishing a European Reference Network for genetic skin diseases.

